# Altered striatal dopamine levels in Parkinson’s disease VPS35 D620N mutant transgenic aged mice

**DOI:** 10.1186/s13041-020-00704-3

**Published:** 2020-12-01

**Authors:** Sarivin Vanan, Xiaoxia Zeng, Sook Yoong Chia, Katarina Varnäs, Mei Jiang, Ke Zhang, Wuan Ting Saw, Parasuraman Padmanabhan, Wei-Ping Yu, Zhi-Dong Zhou, Christer Halldin, Balázs Gulyás, Eng-King Tan, Li Zeng

**Affiliations:** 1 Neural Stem Cell Research Lab, Research Department, National Neuroscience Institute, Singapore, 308433 Singapore; 2 Department of Clinical Neuroscience, Psychiatry Section, Karolinska Institutet PET Centre, Karolinska Institutet, Karolinska University Hospital Solna, R5:02, 171 76 Stockholm, Sweden; 3 Department of Research, National Neuroscience Institute, 11 Jalan Tan Tock Seng, Singapore, 308433 Singapore; 4 Lee Kong Chian School of Medicine, Nanyang Technological University, Singapore, 636921 Singapore; 5 Animal Gene Editing Laboratory, Biological Resource Centre, A*STAR, Singapore, 138673 Singapore; 6 Institute of Molecular and Cell Biology, A*STAR, 61 Biopolis Drive, Proteos, Singapore, 138673 Singapore; 7 Signature Research Program in Neuroscience and Behavioral Disorders Program, DUKE-NUS Graduate Medical School, Singapore, 169857 Singapore; 8 Department of Neurology, National Neuroscience Institute, SGH Campus, Singapore, 169856 Singapore; 9 Center for Molecular Neuropathology, Lee Kong Chian School of Medicine, Nanyang Technological University, Novena Campus, 11 Mandalay Road, Singapore, 308232 Singapore

**Keywords:** Parkinson’s disease, VPS35 D620N, Striatal dopamine, Transgenic mice, Behavioral assay

## Abstract

Vacuolar protein sorting 35 (VPS35) is a major component of the retromer complex that mediates the retrograde transport of cargo proteins from endosomes to the trans-Golgi network. Mutations such as D620N in the VPS35 gene have been identified in patients with autosomal dominant Parkinson’s disease (PD). However, it remains poorly understood whether and how VPS35 deficiency or mutation contributes to PD pathogenesis; specifically, the studies that have examined VPS35 thus far have differed in results and methodologies. We generated a VPS35 D620N mouse model using a Rosa26-based transgene expression platform to allow expression in a spatial manner, so as to better address these discrepancies. Here, aged (20-months-old) mice were first subjected to behavioral tests. Subsequently, DAB staining analysis of substantia nigra (SN) dopaminergic neurons with the marker for tyrosine hydroxylase (TH) was performed. Next, HPLC was used to determine dopamine levels, along with levels of its two metabolites, 3,4-dihydroxyphenylacetic acid (DOPAC) and homovanillic acid (HVA), in the striatum. Western blotting was also performed to study the levels of key proteins associated with PD. Lastly, autoradiography (ARG) evaluation of [^3^H]FE-PE2I binding to the striatal dopamine transporter DAT was carried out. We found that VPS35 D620N Tg mice displayed a significantly higher dopamine level than NTg counterparts. All results were then compared with that of current VPS35 studies to shed light on the disease pathogenesis. Our model allows future studies to explicitly control spatial expression of the transgene which would generate a more reliable PD phenotype.

## Background

Parkinson’s disease (PD) is one of the most common neurodegenerative disorders globally, with the prevalence of PD being reported to be approximately 1% in people 60 years old and older, and increasing to 1–3% in those 80 years old and older [1]. Individuals with PD display motor symptoms such as resting tremor, bradykinesia, rigidity and postural instability [2]. Nonmotor symptoms may also be present, including cognitive impairment, mood disorders and difficulty swallowing [3]. Although PD is largely an idiopathic disease, approximately 5 to 10% of cases are hereditary, with mutations in at least 13 genes being shown to cause familial PD; one of these is the vacuolar protein sorting 35 (VPS35, PARK17) gene [4, 5].

In previous exome sequencing studies, the D620N mutation in VPS35 has been identified as being pathogenic for late-onset autosomal dominant PD in numerous families from various ethnic backgrounds [6, 7]. The VPS35 gene encodes a key component of the retromer complex, which operates by packaging particular endosomal cargoes into tubules and vesicles and then transporting these cargoes either to the trans-Golgi network or to the plasma membrane [8]. The retromer consists of two subprotein complexes: the cargo-selective complex, which contains a VPS35-VPS29-VPS26 trimer [9], and the membrane deformation complex, which consists of a sorting nexin dimer [10].

The upregulation of wild-type (WT) human VPS35 has been shown to rescue α-synuclein-induced neurodegeneration, and the knockdown of endogenous VPS35 resulted in substantial neuronal loss in the hippocampus of α-synuclein transgenic (Tg) mice. These findings suggest that VPS35 is able to functionally antagonize α-synuclein-mediated neurodegeneration [11]. This result is also supported by the findings of Linhart’s study, in which they determined that the overexpression of VPS35 significantly protects against locomotor deficits observed in mutant LRRK2 flies, while knocking down the expression of VPS35 in dopaminergic neurons causes significant locomotor impairment [12]. Together, these findings indicate that VPS35 plays a protective role in regard to dopamine neurons. However, other studies have also presented evidence that VPS35 can have an adverse effect. In rat primary cortical cultures, the overexpression of human WT or VPS35 D620N induces neuronal cell death, while in a novel viral-mediated gene transfer rat model, the expression of human WT or VPS35 D620N induces substantial degeneration and axonal pathology in substantia nigra (SN) dopaminergic neurons [13].

Hence, the generation of genetically engineered rodent models with the VPS35 D620 mutation could not only overcome the problems inherent in using viral transfection but could also provide a crucial tool for understanding the pathophysiology of VPS35. Ishizu’s group first investigated the VPS35 D620N gene in vivo using D620N knock-in (KI) mice, and they found neither homozygous nor heterozygous VPS35 D620N KI mice had suffered premature death or had developed clear signs of neurodegeneration at up to 15 months of age [14]. These results suggest that the VPS35 D620N allele is still functional and does not cause obvious dopamine neuron loss; they also suggest that the VPS35 D620N allele is a partial loss-of-function allele, and genetic predisposition and age-related alterations in the nigrostriatal dopamine system cooperatively influence the pathogenesis of VPS35.

Here, we generated a novel VPS35 D620N transgenic mouse line by integrating a single copy of a transgene (VPS35-D620N) into the mouse Rosa26 locus under the control of an exogenous CAGGS promoter. This strategy allowed us to study a mouse model in which there is spatial expression of the transgene in a physiologically relevant manner, which would generate a more reliable PD phenotype. Furthermore, we analyzed mice at an even older age (20-months-old) to reveal age-related neurodegeneration in Parkinson’s disease.

## Results

### Generation of VPS35 D620N transgenic mice

**Fig. 1:**
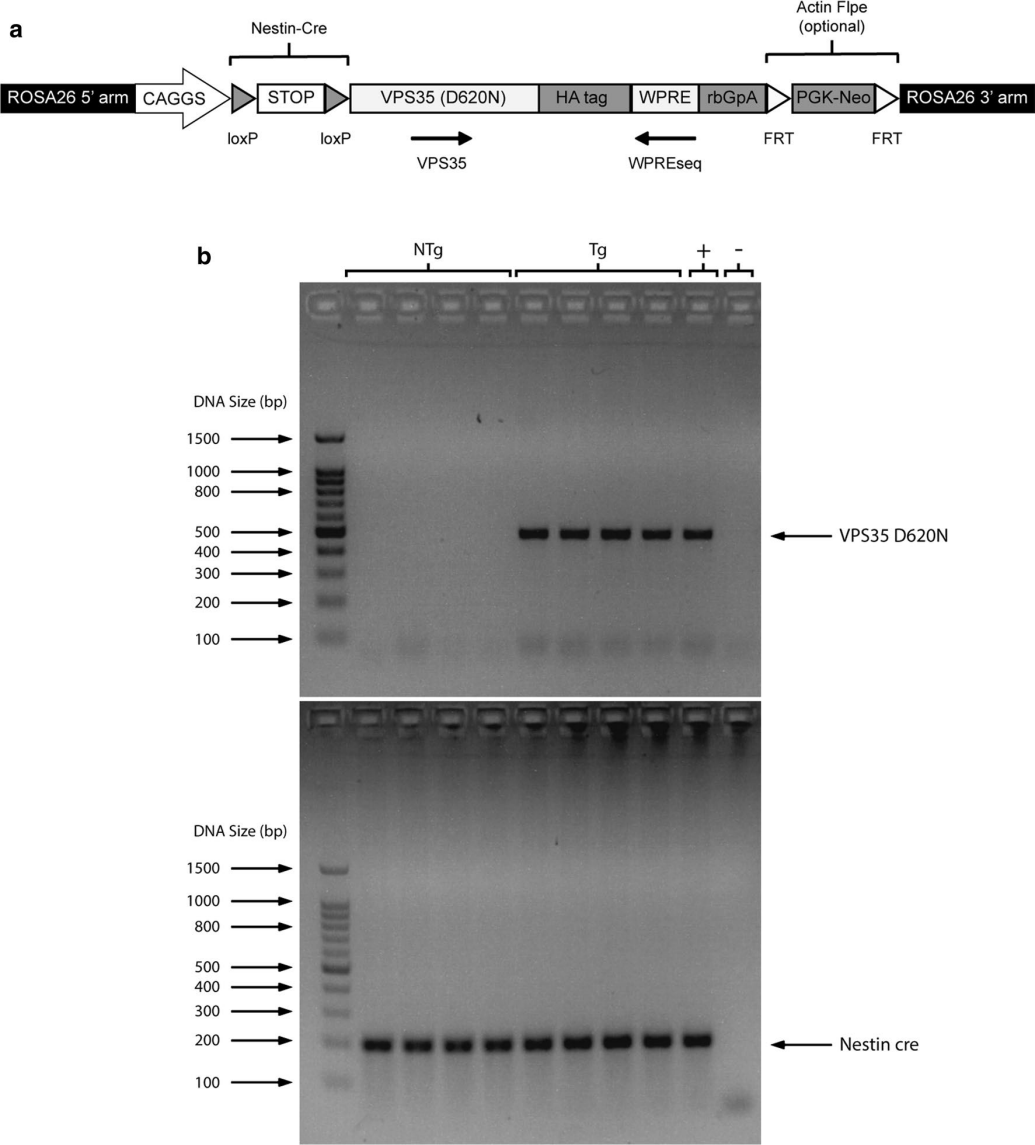
Generation of VPS35 D620N Tg mice. **a** Construct design. The construct designed for VPS35 D620N mouse generation is inserted into the mouse Rosa26 locus under the control of an exogenous CAGGS promoter. A transcriptional termination signal sequence (flanked by two loxP sites) is present between the promoter and transgene coding sequence. Therefore, Cre recombinase is required to derepress the transgene via the excision of the transcriptional termination signal sequence. HA sequence was inserted. **b** PCR testing for VPS35 D620N. VPS35 D620N Tg mice and the positive control display a band at 525 bp, while NTg mice and the negative control do not. All mice are Nestin cre positive and display a band at 200 bp

The generation of this VPS35 D620N mouse model started with the microinjection of the entire VPS35-D620N construct into pronuclei fertilized oocytes of pseudopregnant C57BL/6j females. After establishing germ-line transmission, two founder lines were then backcrossed to produce homozygous founders (C57BL/6J-LSL-VPS35 ^D620N/D620N^). The VPS35-D620N construct itself was inserted into the mouse Rosa26 locus under the control of an exogenous CAGGS promoter (rather than the endogenous Rosa26 promoter). A strong transcriptional termination signal sequence flanked by two loxP sites was inserted between the promoter and the transgene coding sequence, which is, in turn, directly adjacent to a HA tag sequence (Fig. 1A). Under these circumstances (i.e., in the absence of Cre recombinase), the transgene coding sequence is not expressed due to the presence of the transcriptional termination signal sequence. However, in the presence of Cre recombinase, the transcriptional termination signal sequence is excised, which leads to the derepression of the transgene.

Since the homozygous founder line (C57BL/6J-LSL-VPS35^D620N/D620N^) bearing the VPS35 D620N allele can only be expressed in the brain after Cre-mediated excision of the STOP cassette, the homozygous founder lines were crossed with C57BL/6J mice (National University of Singapore Comparative Medicine Centre) first to obtain hemizygous mice (C57BL/6J-LSL-VPS35^D620N/−^). Subsequently, these hemizygous mice were crossed with the B6.Cg-Tg(Nes-cre)1kln/J mouse strain purchased from Jackson Laboratories [15]. The VPS35 D620N transgene can now be specifically expressed in the central and peripheral nervous systems. More importantly, this allows transgenic expression in brain tissue. To confirm the success of the transgene sequence insertion, PCR amplification was performed using designated primers, followed by electrophoresis on a 2% (w/v) agarose gel. All VPS35 D620N transgenic mice showed a clear band at 525 bp, while nontransgenic (NTg) mice had no band. All mice used in this study (both VPS35 D620N NTg and Tg) were Nestin cre positive (Fig. 1b).

### Characterization of transgenic VPS35 D620N mice

**Fig. 2:**
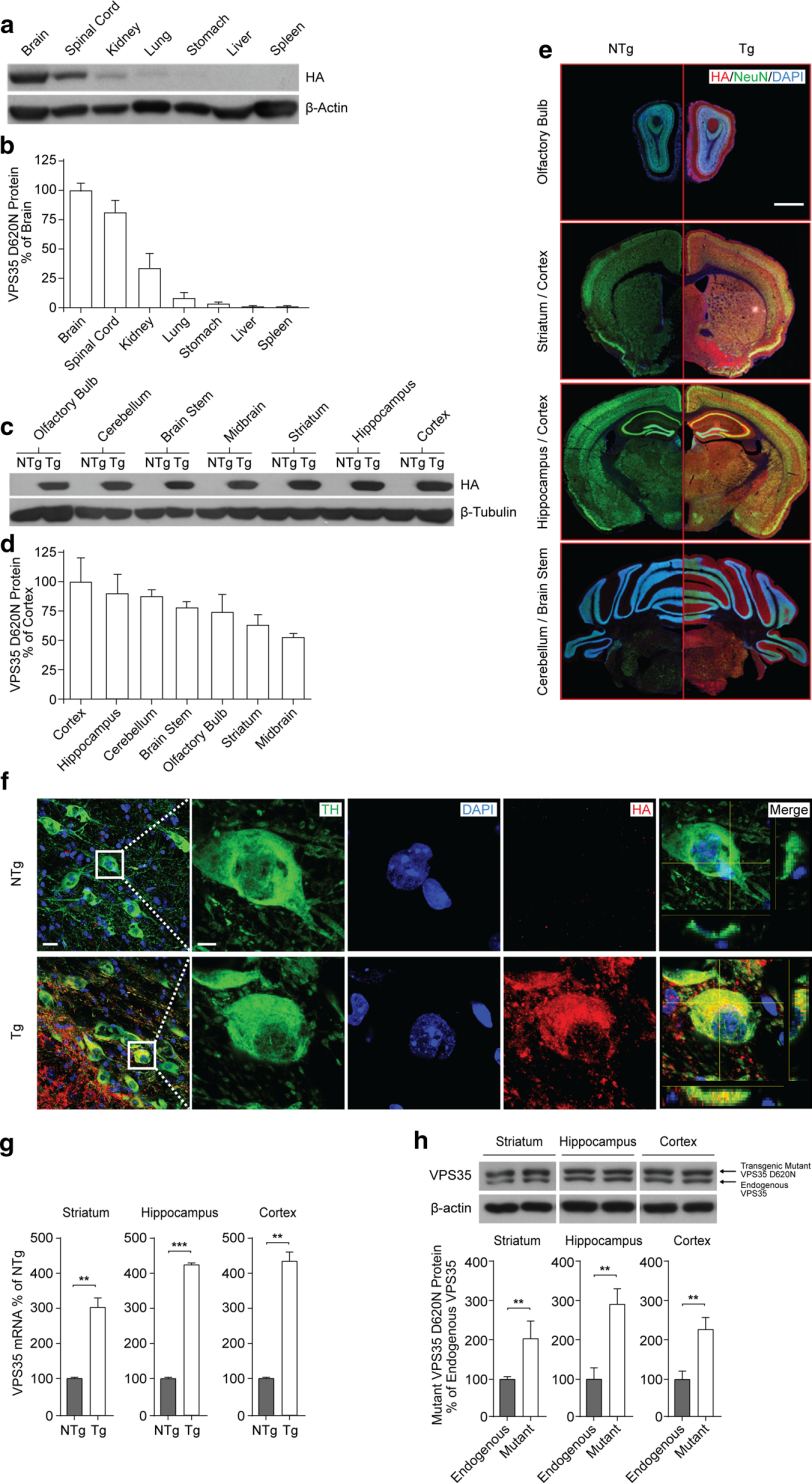
Characterization of 20-months-old VPS35 D620N Tg mice. **a** VPS35 D620N is highly expressed in the CNS. Western blot analysis showed VPS35 D620N is abundantly expressed in the brain, spinal cord and, to a lesser extent, in the kidney, lung and stomach. No expression is observed in the liver or spleen. β-actin is included as a loading control. HA is used to detect VPS35 D620N. **b** Quantification of HA (VPS35 D620N). Expression levels are relative to brain. **c** VPS35 D620N expression in the various brain regions. From the western blot analysis, VPS35 D620N is determined to be expressed in the olfactory bulb, cerebellum, brainstem, midbrain, striatum, hippocampus, and cortex of Tg mice, while no expression is observed in NTg mouse brains. β-tubulin is included as a loading control. **d** Quantification of HA (VPS35 D620N). Expression levels are relative to cortex (most abundant). **e** Immunofluorescence analysis of NTg and Tg mouse brains. Red staining indicates HA (VPS35 D620N), green indicates NeuN, and blue indicates DAPI. VPS35 D620N is abundantly found in Tg mouse brains, specifically in the olfactory bulb, striatum, cortex, hippocampus, cerebellum, and brainstem, but is largely absent in NTg mouse brains. ×4 magnification. Scale bar 1 mm. **f** IHC analysis of TH and HA (VPS35 D620N) in dopaminergic neurons from SN. Green staining indicates TH, blue indicates DAPI, and red indicates HA (VPS35 D620N). There is an absence of HA (VPS35 D620N) in NTg mice, while TH and HA (VPS35 D620N) are colocalized in dopaminergic neurons from Tg mice. Column 1: ×60 magnification. Scale bar 20 μm. Columns 2–5: ×60 magnification. ×5 zoom. Scale bar 5 μm. **g** Evaluation of transgenic mutant VPS35 D620N and endogenous VPS35. QPCR analysis. VPS35 mRNA was extracted from the striatum, hippocampus and cortex of 20-months-old NTg and Tg mice, and quantified (normalized against NTg group). There was a significant increase in VPS35 mRNA in the Tg mice (n = 3) than in age-matched NTg counterparts (n = 3), across the striatum (P = 0.0067), hippocampus (P = 0.00029) and cortex (P = 0.00054); **p < 0.01, ***p < 0.001, Student’s *t*-test. **h** Western blot analysis. VPS35 protein from the striatum, hippocampus and cortex of 20-months-old Tg mice was analyzed. There were two VPS35 bands present for all Tg mice: endogenous VPS35 (lower band) and transgenic mutant VPS35 D620N (upper band). There was a significant increase in transgenic mutant VPS35 D620N compared to endogenous VPS35 across the striatum (P = 0.0014), hippocampus (P = 0.0012) and cortex (P = 0.0031); n = 6. **p < 0.01, Student’s *t*-test

Given that VPS35 D620N mice were crossed with Nestin cre mice, VPS35 D620N (detected by HA antibody) expression was mainly observed in the brain, spinal cord and, to a lesser extent, in the kidney, lung and stomach. No expression was observed in the liver or spleen (Fig. 2a, b). This pattern is consistent with the fact that Nestin cre drives transgene expression in the central nervous system (CNS). To further confirm VPS35 D620N expression in the brain, we fractionated the brain tissue. Western blot results showed that VPS35 D620N protein was expressed in the olfactory bulb, cerebellum, brainstem, midbrain, striatum, hippocampus, and cortex of VPS35 D620N Tg mice, and no expression was observed in NTg mouse brains (Fig. 2c). In terms of the extent of the expression, VPS35 D620N was highly expressed in the cortex, with substantial expression in the hippocampus, cerebellum, brainstem and olfactory bulb, as well; lower levels of expression were observed in the striatum and midbrain (Fig. 2d). Immunofluorescence studies confirmed the western blot results; VPS35 D620N was abundantly detected in Tg mouse brains in various regions (especially in the cortex, hippocampus and cerebellum) but not in NTg mouse brains (Fig. 2e). Additionally, immunohistochemistry analysis of dopaminergic neurons from the substantia nigra showed that VPS35 D620N was colocalized with tyrosine hydroxylase (TH) from Tg mice. This confirms that VPS35 D620N is expressed in dopaminergic neurons (Fig. 2f). Next, we sought to determine if expression levels of transgenic VPS35 D620N were sufficient to overcome endogenous VPS35. Therefore, we used qPCR to analyze VPS35 mRNA from the striatum, hippocampus and cortex of both NTg and Tg mice. We found a significant increase in total VPS35 mRNA in the Tg mice compared to age-matched NTg counterparts, across all three brain regions (Fig. 2g). We also performed western blot analysis to detect endogenous VPS35 protein and transgenic mutant VPS35 D620N protein on tissue from the striatum, hippocampus and cortex of Tg mice. The two proteins were differentiated by the size of the bands with the transgenic mutant VPS35 D620N protein being 1 KDa larger (due to the addition of the HA Tag), and consequently separated from the endogenous VPS35 band. We found a significant increase in transgenic mutant VPS35 D620N compared to endogenous VPS35 in the striatum, hippocampus and cortex (Fig. 2h). Both the qPCR and western blot results demonstrated that the expression levels of VPS35 D620N were sufficient to overcome endogenous VPS35 and is highly expressed in the transgenic VPS35 mutant mice brain.

### Behavior analysis of aged VPS35 D620N transgenic mice

**Fig. 3:**
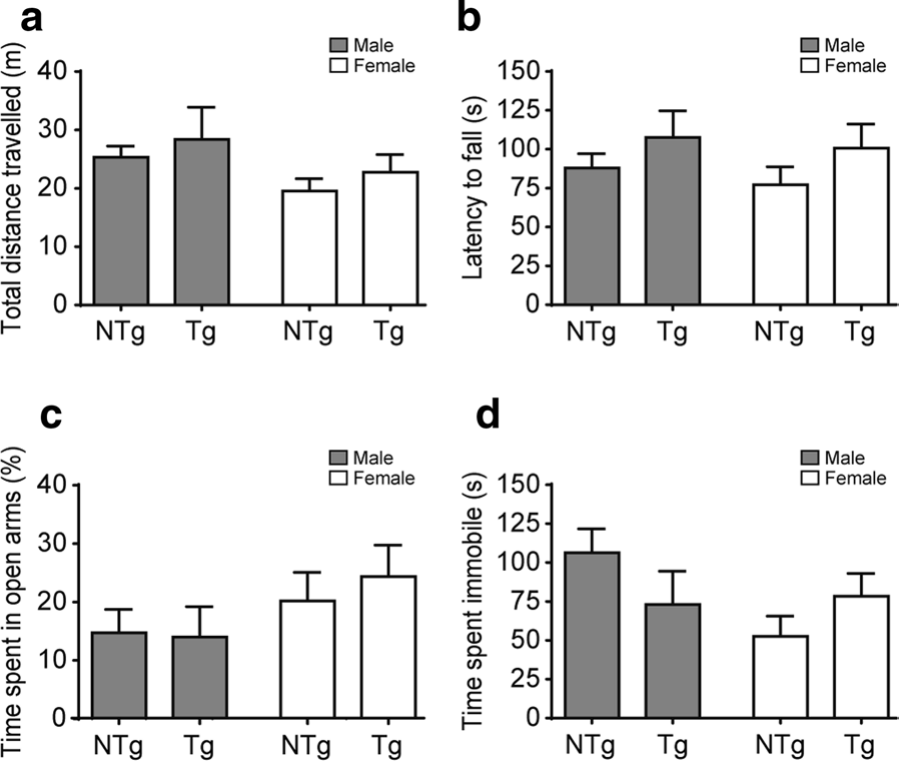
Behavior analysis of 20-months-old VPS35 D620N Tg mice. **a** Open field test. The open field test measures levels of overall movement. There was no significant difference observed in the total distance travelled (m) (mean ± SEM) among all 4 groups: NTg Male (n = 14), Tg Male (n = 7), NTg Female (n = 12), and Tg Female (n = 12); two-way ANOVA with Bonferroni post hoc test. (NTg Male—Tg Male: P = 0.998, NTg Male—NTg Female: P = 0.390, Tg Male—Tg Female: P = 0.988, NTg Female—Tg Female: P = 0.517). **b** Rotarod test. The rotarod test assesses motor coordination ability. There was no significant difference observed in the latency to fall from the rotarod (s) (mean ± SEM) among all 4 groups: NTg male (n = 14), Tg male (n = 7), NTg Female (n = 12), and Tg Female (n = 14). (NTg male—Tg male: P = 0.975, NTg male—NTg Female: P = 0.708, Tg male—Tg Female: P = 0.991, NTg Female—Tg Female: P = 0.637). **c** Elevated plus maze. The elevated plus maze tests for anxiety. There was no significant difference observed in the percentage of time spent in the open arms (mean ± SEM), among all 4 groups: NTg male (n = 15), Tg male (n = 4), NTg Female (n = 10), and Tg Female (n = 13). (NTg male—Tg male: P = 0.999, NTg male—NTg female: P = 0.849, Tg male—Tg female: P = 0.693, NTg female—Tg female: P = 0.931). **d** Tail suspension test. The TST is a test for depression. There was no significant difference observed in the time spent immobile (s) (mean ± SEM), among all 4 groups: NTg male (n = 15), Tg male (n = 7), NTg female (n = 12), and Tg female (n = 13). (NTg male—Tg male: P = 0.542, NTg male—NTg female: P = 0.063, Tg male—Tg female: P = 0.997, NTg female—Tg female: P = 0.632)

Because motor symptoms are considered the hallmark of PD in human patients [16, 17], we first conducted an assessment of general movement ability with the open field test, and motor coordination ability with the accelerated rotarod test in both male and female mice aged 20-months-old. There was a trend toward Tg mice showing increased total distance travelled in the open field (Fig. 3a) and a slightly longer latency to fall from the rotarod (Fig. 3b), but no significant difference between NTg and Tg mice was observed.

Since dopamine-related mood disorders, gut dysfunction, attention and cognition deficits, and memory loss have been increasingly shown to be important features of Parkinson’s disease [18–20], NTg and Tg littermates were further tested in the elevated plus maze and tail suspension test (TST) to evaluate levels of anxiety-like behaviors and depressed states in the animals, respectively. We did not observe any tendency for Tg mice to be more anxious than the NTg mice for both males and females. Both spent a similar percentage of time in the open arms, with no significant difference detected (Fig. 3c). A slightly shorter immobile time was observed in Tg mice in the TST, which suggested that Tg mice made more attempts to escape (less depressed), but there was no significant difference between Tg and NTg mice (Fig. 3d).

Interestingly, there were no discernible sex-specific differences observed in the behavioral assay. Lastly, the body weight of mice was analysed in order to determine if there were variations that could have affected the behavior results; no significant differences were observed (Additional file 1: Fig. S1A). Overall, our behavioral test results indicated that there were no significant differences between Tg and NTg mice in terms of general levels of movement, motor coordination, anxiety, and depression for both male and female mice.

### Histology and stereological quantification of tyrosine hydroxylase neurons in the substantia nigra

**Fig. 4:**
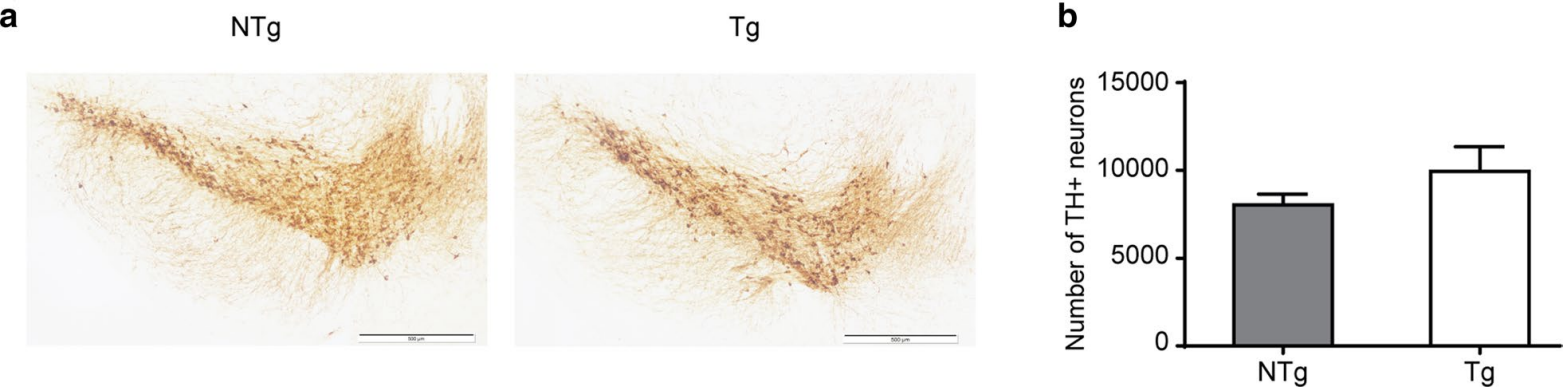
Histology and stereological quantification of TH neurons in the substantia nigra (SN). **a** Representative images of TH-positive neurons in the NTg (left) and Tg (right) SN from 22-months-old mice. Anti-TH antibody was used for staining. ×10 magnification. Scale bar 500 µm. **b** Stereological counting of TH-positive neurons in the NTg and Tg mouse SN (mean ± SEM). 7 brain sections from each mouse were used for the unbiased stereological analysis. ×63 magnification. No significant difference (P = 0.136) in the number of TH-positive neurons in the SN was observed between NTg mice (n = 5) and Tg controls (n = 5); Student’s *t*-test

Selective loss of dopaminergic neurons in the substantia nigra (SN) is believed to be initiated long before the onset of PD. To determine whether these characteristic neuropathological changes can be seen in the VPS35 D620N Tg mouse brain, we conducted DAB staining using an antibody against tyrosine hydroxylase (TH). The total number of TH-positive neurons in the SN was quantified in the brains of NTg control and VPS35 D620N Tg mice using unbiased stereological analysis (Fig. 4a). We found no significant difference in the number of TH-positive cells in the SN of transgenic mice and NTg age-matched controls (Fig. 4b).

### HPLC analysis of dopamine and its metabolites in the striatum

**Fig. 5:**
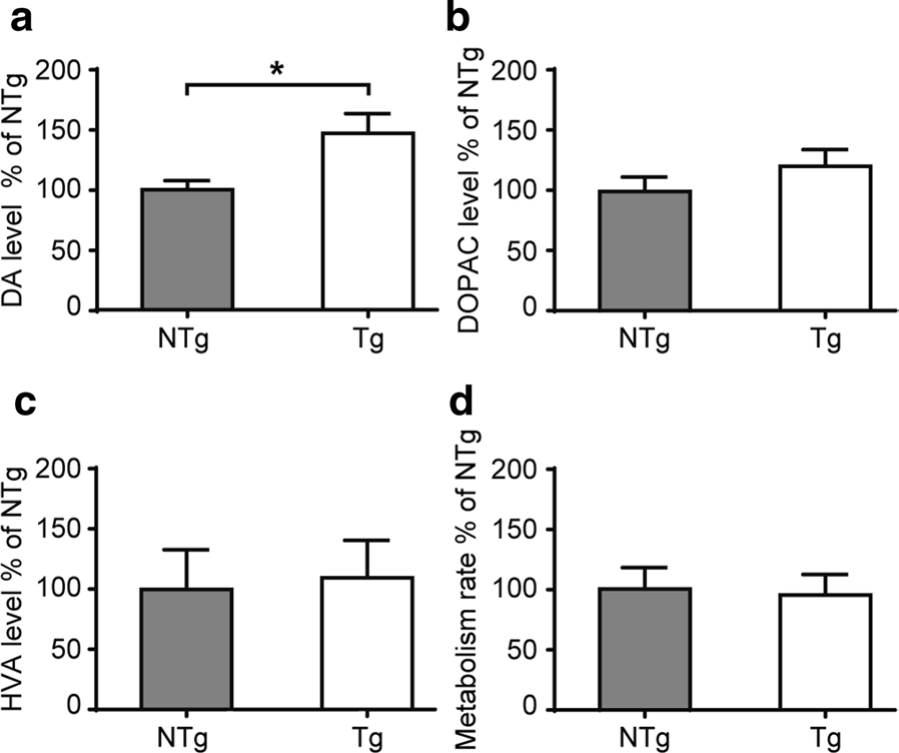
HPLC analysis of DA and its metabolites in the striatum of 22-months-old mice. **a** HPLC analysis of DA levels in the striatum normalized to the NTg group (mean ± SEM). DA levels were significantly (P = 0.028) higher in Tg mice (n = 7) than in age-matched NTg counterparts (n = 9); *p < 0.05, Student’s *t*-test. **b** HPLC analysis of DOPAC levels in the striatum normalized to the NTg group (mean ± SEM). DOPAC is a DA metabolite. No significant difference (P = 0.410) was observed in DOPAC levels between NTg (n = 9) and Tg mice (n = 7). **c** HPLC analysis of HVA levels in the striatum normalized to the NTg group (mean ± SEM). HVA is another metabolite of DA. No significant difference (P = 0.701) was observed in HVA levels between NTg (n = 5) and Tg mice (n = 6). **d** Metabolism rate normalized to the NTg group (mean ± SEM). The (DOPAC + HVA)/DA ratio is used as an indicator of the rate of DA turnover. There was no significant difference (P = 0.712) observed between NTg (n = 9) and Tg mice (n = 7). Student’s *t*-test

Current studies showed discrepancies on whether the VPS35 D620N mutation leads to neurochemical changes in the dopaminergic terminals of the mutant mice [14, 21, 22]; therefore, we measured the concentrations of dopamine (DA), as well as the concentration of its two metabolites, 3,4-dihydroxyphenylacetic acid (DOPAC) and homovanillic acid (HVA), in striatal lysates. HPLC analysis revealed that there was a significant increase in DA levels, while no significant changes in DOPAC and HVA levels were observed in VPS35 D620N Tg and age-matched NTg mice (Fig. 5a–c). The DOPAC + HVA/DA ratio can be used as an indicator of the rate of DA turnover, and there was no significant difference in this ratio between the two groups (Fig. 5d), suggesting that the mutation did not affect the metabolism of DA.

Furthermore, we studied the interaction between VPS35 D620N and exogenous oxidative challenge to determine if VPS35 D620N confers vulnerability towards exogenous oxidative stress. Here, SH-SY5Y cells were transfected with VPS35 D620N and subjected to 24 h of 20 µM H_2_O_2_ treatment [23]. Two parameters were analyzed in this study, cell death (Trypan Blue Assay) and the level of HVA and DOPAC detected by HPLC. As expected, a significant increase in cell death was observed in both the control and VPS35 D620N transfected cells after 24 h of H_2_O_2_ treatment; however, there was no significant difference between VPS35 D620N and control transfected cells after 24 h of H_2_O_2_ treatment (Additional file 2: Fig. S2A). Additionally, upon HPLC analysis, no significant difference was observed in HVA levels (Additional file 2: Fig. S2B) and DOPAC levels (Additional file 2: Fig. S2C) across all 4 groups. Our results suggest that VPS35 D620N has no vulnerability towards exogenous oxidative challenge.

### Western blot analysis of PD related proteins in the striatum

**Fig. 6:**
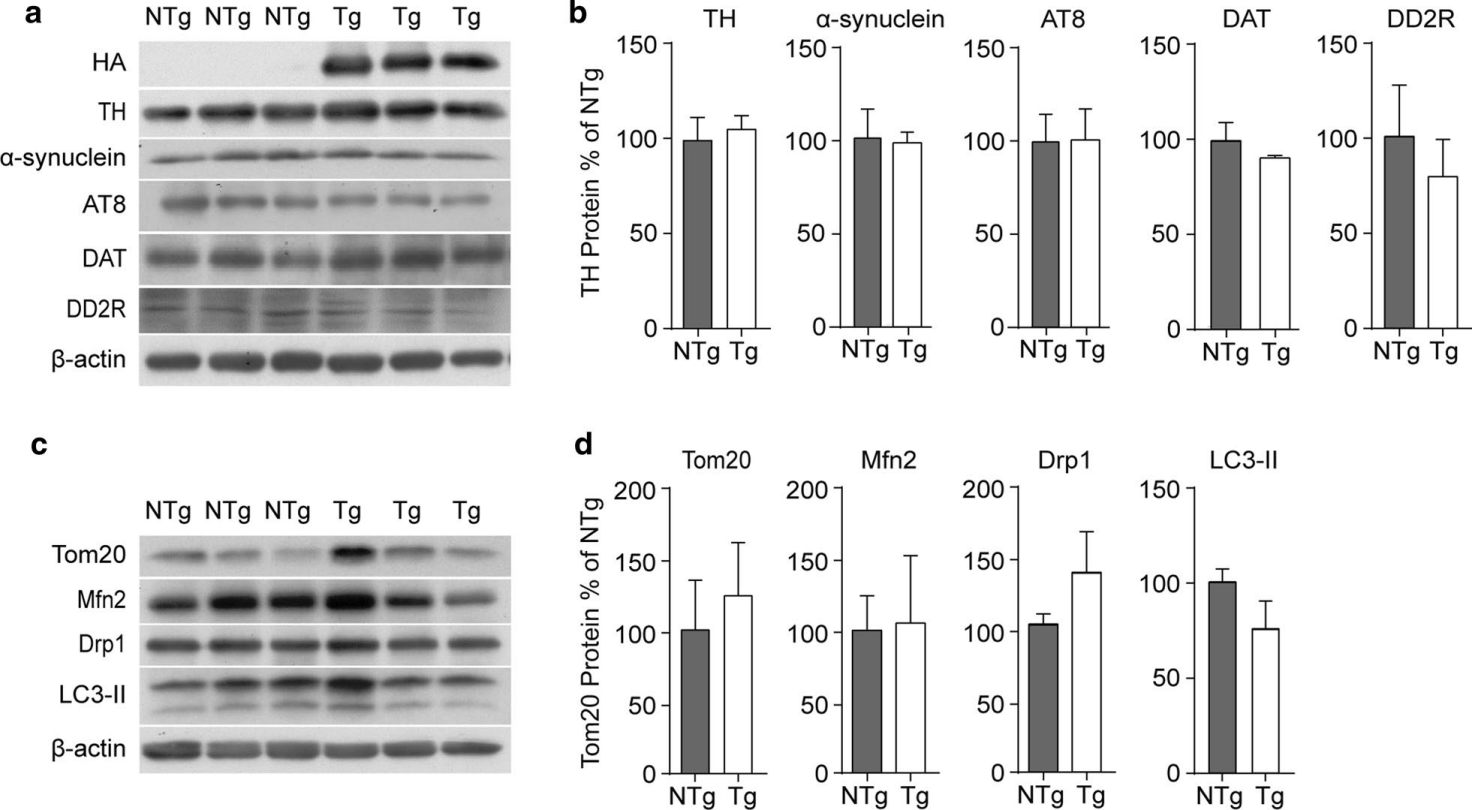
Western blot analysis of PD related proteins in the striatum. **a** Striatal samples from 22-months-old NTg mice (n = 3) and Tg mice (n = 3) were tested for a variety of PD associated proteins: TH, α-synuclein, Phosphor-Tau (AT8), DAT and DD2R by Western blot. β-actin was used as a loading control. **b** Quantification analysis of proteins detected (mean ± SEM). There was no significant difference in the relative expression of TH (P = 0.500), α-synuclein (P = 0.886), AT8 (P = 0.831), DAT (P = 0.407) and DD2R (P = 0.532). Student’s *t*-test. **c** Striatal samples were tested for the mitochondrial proteins Tom20, Mfn2 (Mitofusin-2), and Drp1, and the autophagy-related protein LC3-II by Western blot. β-actin was used as a loading control. **d** Quantification analysis of proteins detected (mean ± SEM). There was no significant difference in the relative expression of Tom20 (P = 0.662), Mfn2 (P = 0.927), Drp1 (P = 0.283) and LC3-II (P = 0.210). n = 3. Student’s *t*-test

To further characterize VPS35 D620N mice, we conducted western blotting to assess the PD-associated pathogenic genes in the striatum. All VPS35 D620N Tg mice showed an anti-HA positive band, while NTg mice did not (Fig. 6a). The loss of VPS35 function has been associated with the reduced lysosomal degradation of α-synuclein and increased levels of AT8 (Phospho-Tau Ser202/Thr205) [22], while the overexpression of the VPS35 D620N protein is linked to a loss of DA neurons in the substantia nigra pars compacta (SNpc) [5, 13, 22, 24]. Therefore, we first measured the expression levels of TH, α-synuclein, and AT8 in the striatum [25] by western blot. We found that there were no significant differences in TH, α-synuclein and AT8 expression between VPS35 D620N Tg and NTg mice (Fig. 6a, b). Since the retromer is important for the normal functioning of neurons with neurotransmitter receptors acting as cargo molecules of the retromer [26–28], we next analyzed the expression levels of the striatal dopamine transporter (DAT) and dopamine D2 receptor (DD2R), which are related to dopamine transmission at the pre- and postsynapse of dopamine neuron terminals, respectively. It should also be noted that DAT and the dopamine D1 receptor (DRD1) have been reported to be retromer-dependent for synapse membrane trafficking and recycling [28, 29]. The western blot results showed no significant differences in DAT and DD2R expression (Fig. 6a, b).

Mitochondrial dysfunction has persistently been implicated in the pathogenesis of both familial and idiopathic PD [30]. VPS35, in particular, has been implicated in mitochondrial function through its reported role in mediating vesicle transport from mitochondria to peroxisomes or lysosomes by regulating the formation of mitochondrial-derived vesicles (MDVs) [31, 32]. Therefore, we subsequently tested for the mitochondrial proteins Tom20, Mitofusin-2 (Mfn2) and Drp1. Tom20 acts as an import receptor for mitochondrial proteins [33], while Mitofusin-2 is involved in mitochondrial fusion, trafficking and turnover [34]. Drp1 participates in outer mitochondrial membrane fission [35]. Lastly, we analyzed the expression level of LC3-II which is related to autophagy [36]. We found that there were no significant differences in Tom20, Mfn2, Drp1 and LC3-II expression across VPS35 D620N Tg and NTg mice (Fig. 6c, d).

### ***Autoradiographic evaluation of [***^***3***^***H]FE-PE2I binding to DAT in the striatum***

**Fig. 7:**
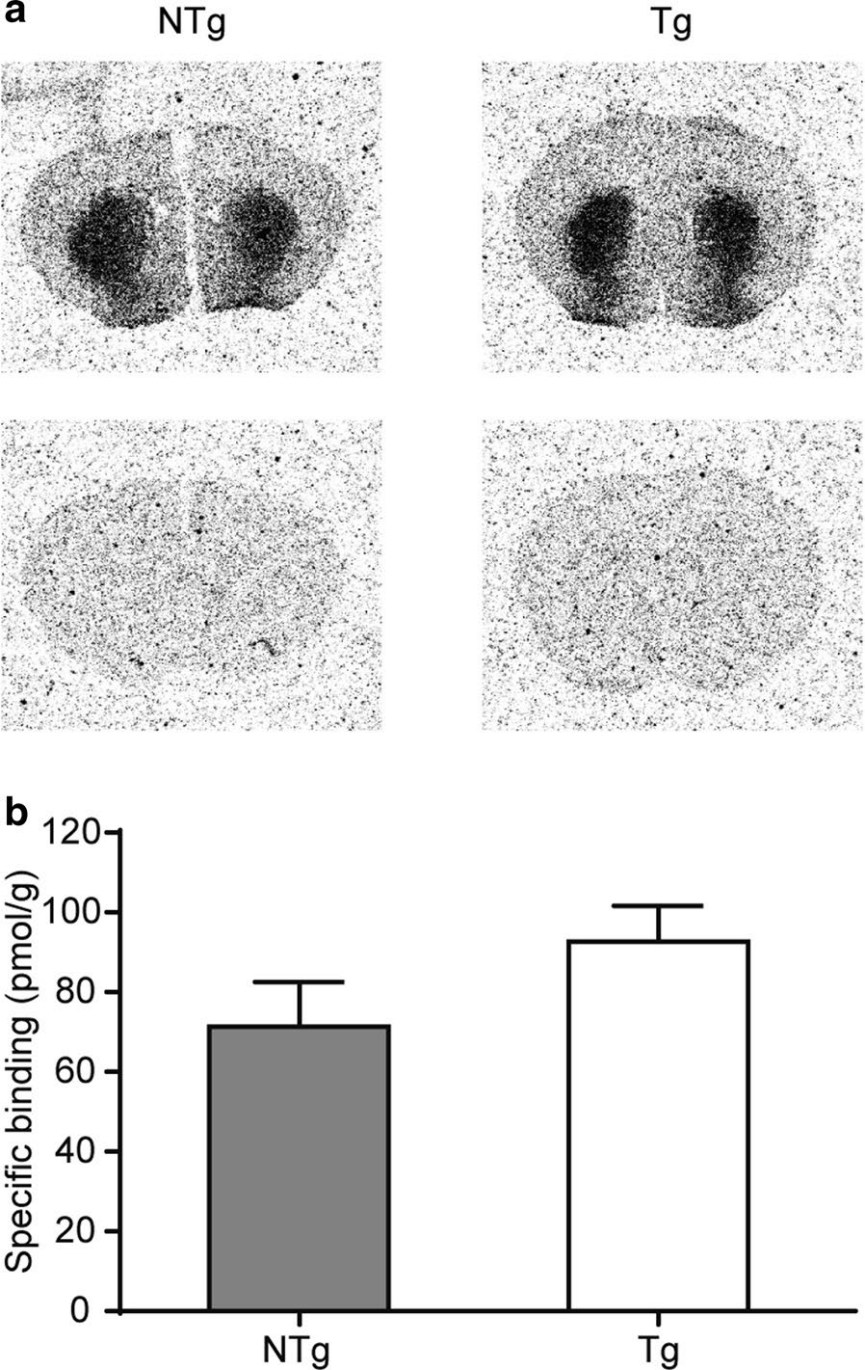
ARG evaluation of [^3^H]FE-PE2I binding to DAT in the striatum. **a** DAT binding in striatum. [^3^H]FE-PE2I binds to DAT with high potency. This binding is inhibited by the DAT inhibitor GBR12909. Thus, in vitro ARG using radiolabeled analogues of PE2I provides detailed qualitative evidence of the binding to DAT. Here, ARG is performed on striatal sections of 22-months-old NTg and Tg mice. Images show total binding of the radiolabeled tracer [^3^H]FE-PE2I (upper panel), and nonspecific binding in adjacent brain sections that were co-incubated in the presence of GBR12909 (lower panel). **b** Quantitative analysis of the radioactivity level in the striatum of DAT binding (pmol/g) between NTg and Tg mice (mean ± SEM). There was no significant difference (P = 0.216) in striatal DAT binding between NTg and Tg mice. n = 3. Student’s *t*-test

PE2I binds to DAT with high potency but has very low affinities for serotonin and noradrenaline transporters. PE2I binding can be displaced by the dopamine transporter inhibitor GBR12909. Thus, in vitro autoradiography (ARG) using radiolabeled analogues of PE2I has provided detailed maps of the binding density of dopamine transporters in the brain [37]. To further confirm the western blot results for DAT detection (Fig. 6a, b), in vitro ARG was performed on striatal sections from NTg and Tg mice. Specific binding to DAT was represented as the difference between the total binding of the radiolabeled tracer [^3^H]FE-PE2I, and nonspecific binding determined in adjacent brain sections that were co-incubated in the presence of non-radiolabeled GBR12909. After quantitative analysis of the radioactivity level in the dorsal part of the striatum sections, our results showed no significant difference in DAT binding between NTg and Tg mice (Fig. 7a, b). This result is consistent with the western blot findings.

## Discussion

To address the discrepancies in terms of results and methodologies in the VPS35 studies thus far, we first generated transgenic VPS35 D620N mice and then subjected them to a series of behavioral tests (focusing on levels of overall movement, motor coordination, anxiety, and depression). Next, DAB staining, HPLC, western blot and ARG were performed in succession to study the differences between the VPS35 D620N Tg mice and age-matched controls.

The VPS35 D620N model was based on inserting the VPS35-D620N construct into the mouse Rosa26 locus under the control of an exogenous CAGGS promoter (Fig. 1a). In contrast to animal models generated via the conventional random integration method, which often leads to variations in transgene expression (due to random integration), this VPS35 D620N model allowed genomic integration of VPS35-D620N as a single copy and at a known genomic locus [38]. Additionally, the Nestin-cre system was employed to specifically express VPS35 D620N in the central and peripheral nervous systems, which makes it vastly more relevant to the study of PD.

Behavioral validation in our VPS35 D620N Tg mice showed no difference in terms of motor function. They travelled slightly longer distances in the open field (Fig. 3a) and exhibited longer latencies before falling from the rotarod (Fig. 3b) but the difference was not significant; Tg mice also did not show any anxiety or depression symptoms in the elevated plus maze (Fig. 3c) and tail suspension test (Fig. 3d), respectively. We conclude that our VPS35 D620N Tg mice did not show any evidence of motor or emotional deficits. These results are in line with those of Ishizu’s VPS35 D620N KI mice; their homozygous KI mice did not develop any PD-like phenotypes at up to 17-months-old [14]. These findings are also in agreement with Chen’s and Cataldi’s studies, which have likewise observed no significant behavior phenotypes in adult and aged VPS35 D620N KI mice [21, 22] (Table 1).

**Table 1:** Comparison of various VPS35 animal models studies

Paper		Impaired striatal dopamine release in homozygous Vps35 D620N knock-in mice (Ishizu et al*.* 2016)	Altered dopamine release and monoamine transporters in Vps35 p.D620N knock-in mice (Cataldi et al*.* 2018)	Parkinson’s disease-linked D620N VPS35 knockin mice manifest tau neuropathology and dopaminergic neurodegeneration (Chen et al*.* 2019)	Parkinson’s disease-linked mutations in VPS35 induce dopaminergic neurodegeneration (Tsika et al*.* 2014)	Parkinson’s disease genes VPS35 and EIF4G1 interact genetically and converge on α-synuclein (Dhungel et al*.* 2015)	Our study
Age		Aged, 17.5 months VPS35 and VPS35 D620N mice	Adult VPS35 and VPS D620N mice	Adult and aged, 3–24 months VPS35 and VPS35 D620N mice	VPS35 and VPS35 D620N virus injected into adult wildtype rat	VPS35 virus injected into adult α-synuclein mice	Aged, 20 months VPS35 D620N mice
Sex		Male and female	Male	Male and female	N.A	N.A	Male and female
Generation		Mouse (KI)	Mouse (KI)	Mouse (KI)	Virus injected rat	Virus injected mouse	Mouse (Tg)
Biochemistry results	Western blot	No change in DRP1, LC3-II, Cathepsin D, Tom20, VDAC1, MUL1, α-synuclein, Parkin, LAMP1, GM130, and EEA1	No change in TH ↑ VMAT2 ↓ DAT in VPS35 D620N^female^KI mice	No change in VPS35 and α-synuclein	No change in VPS35, Sortilin, SorLA, Cathepsin D, and TH in VPS35 and VPS35 D620N virus injected rats	N. A	No change in Drp1, LC3-II, Tom20, TH, DD2R, DAT, Mfn2, α-synuclein, and AT8 in VPS35 D620N Tg mice
	Immuno-histochemical staining	N.A	↓ DAT integrated density ↓ DAT area in VPS35 D620N KI mice	↑ AT8 ↑ Tau5 ↑ MT1 ↑ APP + spheroids ↑ degenerating neuritic processes in VPS35 D620N KI mice	↑ TUNEL assay = ↑ neuronal cell death ↓ neurite outgrowth ↑ neuronal vulnerability to cellular stress in VPS35 and VPS35 D620N virus injected rats	↑ NeuN ↓ α-synuclein ↓ GFAP ↓ neuroinflammation in VPS35 virus injected α-synuclein mice	N. A
	HPLC	No change in DA, DOPAC, and HVA	No change in DA, DOPAC, and HVA ↑ (DOPAC + HVA)/DA ratio in VPS35 D620N	No change in DA, DOPAC, and HVA	N.A	N.A	↑ DA in VPS35 D620N Tg No change in DOPAC, HVA, and (DOPAC + HVA)/DA ratio
	Stereological analysis of TH + neurons	No significant difference observed	No significant difference observed	↓ in VPS35 D620N KI mice	↓ in VPS35 D620N injected rats	N.A	No significant difference observed
Behavior results		Open-field test: no difference	Open-field, rotarod, and cylinder test: no difference	Open-field, rotarod, and gait analyses: no difference	Cylinder test: no difference between control, VPS35, and VPS35 D620N	N.A	Open-field, rotarod, and elevated plus maze: no difference

Differences in the results and methodologies are examined then compared with our study, particularly in terms of biochemistry results (western blot, immunohistochemical staining, HPLC, stereological analysis of TH + neurons) and behavior results

In our VPS35 D620N Tg mice, higher dopamine content in the striatum was observed in transgenic mice compared with age-matched non-transgenic control mice (Fig. 5a). There was a relatively similar level of the dopamine metabolites DOPAC (Fig. 5b) and HVA (Fig. 5c), as well as the dopamine turnover rate (Fig. 5d) between Tg and NTg mice. This suggested that the higher dopamine content in the striatum was not due to the interruption of dopamine breakdown. These results are contradictory to the studies that found no significant difference in DA levels between VPS35 D620N KI mice and controls [14, 21] However, the lack of any significant difference in DOPAC and HVA levels are consistent throughout these studies [14, 21] and ours (Table 1). We speculate that the mutated form of VPS35 protein is still functional to a certain degree in vivo, which is supported by Ishizu’s results [14]. In their homozygous, as well as heterozygous VPS35 D620N KI mice that were up to 17-months-old, VPS35 expression levels, VPS35 retromer complex formation, and mitochondrial functions were not affected. No obvious dopamine neuron degeneration or increased α-synuclein deposition was observed. Importantly, they show that a single copy of the mutated VPS35 D620N allele could rescue the embryonic lethality of VPS35 deletion 1 (Del1) mice, which highly suggests that the mutated form of the VPS35 protein is still functional to a certain degree. In should be noted that in our VPS35 D620N Tg mice, the endogenous VPS35 gene still exists; therefore, the VPS35 protein may still function normally in cells. The accumulation of this endogenous form of VPS35 along with the overexpressed mutated form (VPS35 D620N protein) may, therefore, be responsible for the protective effects against dopamine neuronal damage; the overexpression of VPS35 could rescue locomotor deficits by rescuing dopamine neuron degeneration [11, 13]. To confirm this hypothesis, VPS35-related functions such as retromer assembly, membrane protein trafficking and mitochondrial activity, as well as neuronal cell survival, can be tested using in vitro cultures of neurons isolated from VPS35 D620N mice in future.

Biochemistry analysis showed that the level of α-synuclein in the striatum of Tg mice was not increased (Fig. 6b), suggesting a lack of accumulation of α-synuclein, which is a hallmark of PD pathogenesis. This finding further suggests that it was unlikely that our VPS35 D620N Tg mice developed PD neuropathy, and the literature reports that the overexpression of VPS35 D620N impairs the degradation of α-synuclein, which eventually leads to a loss of dopamine neurons [11, 24, 39, 40]. However, our finding is in line with Chen’s study which found no significant alternations in α-synuclein levels in the brains of 13-months-old VPS35 D620N KI mice [22], and Ishizu’s study which also found no significant difference in α-synuclein in their VPS35 D620N KI mice [14] (Table 1). DAT and DD2R play important roles in dopamine neuron transmission as dopamine transporters and receptors localizing in presynaptic and postsynaptic membranes, respectively. DAT recaptures excess dopamine and transports it back into dopamine neurons to be recycled and broken down. However, the mechanism for controlling membrane expression levels, and recycling DAT and postsynaptic dopamine receptors is not fully understood. Only recently have papers reported that both DAT and DD1R endocytic recycling require intact retromer complexes [28, 29]. The overexpression of VPS35 could increase DD1R membrane expression levels but not total protein levels of DD1R [28], and higher striatal dopamine levels were often related to DAT dysfunction [41, 42]. Our results showed no significant change in DD2R levels in the striatum of VPS35 D620N Tg mice (Fig. 6b), which is in line with the above findings. Certainly, we cannot rule out if the mechanism of the downregulation of DAT expression is due to compensatory or direct effects related to the overexpression of VPS35 in this study. Lastly, in line with the western blot results for DAT expression (Fig. 6b), our in vitro ARG results suggested a similar DAT binding function between VPS35 D620N Tg mice and age-matched controls (Fig. 7b).

## Conclusion

In conclusion, we generated a novel VPS35 D620N transgenic mouse model by adopting a strategy that allows the expression of the transgene in mice in a spatial manner. An array of behavioral (motor and non-motor) and biochemistry analyses (stereological analysis of TH-positive neurons, HPLC analysis of DA, DOPAC and HVA levels, autoradiographic evaluation of [^3^H]FE-PE2I binding to DAT, and western blot studies on key proteins associated with PD) was conducted. We found an increased dopamine content in VPS35 D620N Tg mice compared to NTg controls. Our model allows future studies to explicitly control spatial expression of the transgene which would generate a more reliable PD phenotype.

## Materials and methods

### Animals

All mice were housed in standard cages in a temperature controlled (22 ± 2 °C) and pathogen-free room under diurnal conditions (12 h light/dark cycle) with food and water available ad libitum. All animal procedures were in accordance with the *guidelines on the Care and Use of Animals for Scientific Purposes* developed by the National Advisory Committee for Laboratory Animal Research (NACLAR) and approved by Institutional Animal Care and Use Committee (IACUC) of the National Neuroscience Institute.

### Transgenic mouse production

A targeting vector was designed to contain the VPS35 D620N mutation gene downstream of a *loxP*-flanked stop sequence (LSL). C57BL/6J-LSL-VPS35^D620N/−^ mice were subsequently generated by microinjecting this entire construct, which was inserted into the *ROSA26* locus, into the pronuclei fertilized oocytes of pseudopregnant C57BL/6j females. After establishing germ-line transmission, two founder lines were first backcrossed to produce homozygous founders (C57BL/6J-LSL-VPS35^D620N/D620N^).

The homozygous founder line (C57BL/6J-LSL-VPS35^D620N/D620N^) bearing the VPS35 D620N allele can only be expressed after Cre-mediated excision of the STOP cassette. Hence, the homozygous founder lines were crossed with C57BL/6J mice (National University of Singapore Comparative Medicine Centre) first to obtain hemizygous mice (C57BL/6J-LSL-VPS35^D620N/−^). Then, the hemizygous mice were crossed with the C57BL/6J-Nestin-cre mouse strain purchased from Jackson Laboratories. Progeny, including C57BL/6J-VPS35^D620N/−^ (designed as the Tg mice) and C57BL/6J-VPS35 ^−/−^ (designed as the NTg mice) mice, were genotyped for the presence of the VPS35 D620N allele using specific PCR primers (VPS35s5: 5′-TTGCGCTTGCGGCGGGAATCACCAG-3′ and VPS35gt1: 5′-ACACTGGAGCATTTGCGCTTGCGGC-3′). Similarly, sense primers (5′- CCGGTGAACGTGCAAAACAGGCTCTA-3′) and anti-sense primers (5′-GATTAACATTCTCCCACCGTCAGT-3′) were used to detect the presence of the Nestin cre gene sequence.

### Neuropathological characterization of transgenic mice

VPS35 D620N Tg mice and age-matched control NTg mice were anesthetized with ketamine/xylazine (100/10 mixture; 0.1 mg g^−1^ body weight, intraperitoneal injection) and perfused with ice-cold PBS followed by 4% paraformaldehyde in PBS. Mouse brains and selected organs were collected, dissected and soaked in fixative for 24 h and then transferred into 30% sucrose buffer for 2–3 days until they sank down to the bottom of the tube. Subsequently, brain samples were sliced into 30 µm coronal section series on a Micromcryostat (Leica CM3050S) at − 20 °C. Immunofluorescence staining was performed on free-floating sections that were then stained in a solution containing 0.1% BSA, 0.1% Triton X-100 and the rabbit Anti-HA tag [HA. C5] antibody (Abcam#ab18181), and rabbit anti-NeuN antibody (Millipore#ABN78). After a series of 0.1 M PBS washes, sections were stained using the same blocking solution as above and Alexa Fluor 488 goat anti-rabbit and Alexa Fluor 555 donkey anti-mouse secondary antibodies (Inventrogen).

### Immunohistochemistry (IHC) staining

35 µm coronal brain sections of NTg and Tg mice were washed with Dulbecco’s phosphate-buffered saline (PBS) pH 7.4 for 5 min. They were blocked in 1% BSA including 0.1% Triton X-100 at room temperature for 30 min, which was followed by incubation of the primary antibodies overnight at 4 °C. The following primary antibodies were used in this study (1:200): rabbit anti-HA (Cell signaling technology, #3724S) and mouse anti-TH (Millipore, #MAB318). Next, brain sections were washed with PBS 3 times, for 5 min each time, and incubated with secondary antibodies (1:400): goat anti-mouse Alexa Fluor 488 (Invitrogen, #A-11001) and goat anti-rabbit Alexa Fluor 555 (Invitrogen, #A-21428). Brain sections were also counterstained with the fluorescent nuclear dye 4′,6-diamidino-2-phenylindole dihydrochloride (DAPI, Sigma-Aldrich, #B2261). Subsequently, brain sections were washed with PBS again (3 times, 5 min each time). Finally, brain sections were washed, dried and mounted with DAKO Fluorescence Mounting Medium.

### Stereology cell counting

Using the previously collected mouse brain samples, 35 µm coronal sections were cut (Leica CM1950), and free floating sections were incubated overnight at room temperature with rabbit anti-TH primary antibody (Novus Cat. NB300-109), followed by incubation for 1 h with secondary antibody using the Elite rabbit IgG kit (vector laboratory PK-6101) [43]. The DAB kit (Vector laboratory PK-4100) was used for color development.

The number of TH positive dopamine neurons in the SN was estimated using unbiased stereological counting. The SN was delineated from 1.70 to 3.88 mm posterior to bregma using the Allen brain atlas [44]. A total of 7 coronal sections from the midbrain of each mouse were used for quantification (1 section from every 5 serial coronal sections across the midbrain was used); representative pictures containing TH^+^ neurons in the SN were taken using the 10 × magnification objective lens of an Olympus IX83 microscope. Scale bar, 500 µm. The number of TH^+^ neurons in each section was stereologically quantified using Stereologer 2000™, at 63 × magnification. The 7 sections from each mouse were then used to compare the number of TH^+^ neurons in Tg and NTg mice (n = 5 for Tg, n = 5 for NTg).

### HPLC measurement of DA, DOPAC and HVA

The brains of VPS35 D620 transgenic and age-matched NTg mice were quickly removed and washed with ice-cold phosphate-buffered saline (PBS). The striatum of the right hemispheres were removed. Mice striatal tissue was lysed in 0.5 M perchloric acid. The levels of DA, DOPAC and HVA of the tissue were measured by Reversed-phase UltiMate 3000 HPLC system (Germany) with ECD detector and a reversed-phase column (DBS HYPERSIL C18, 25 cm × 3.0 mm, 5 μm particle size, 130 Å pore size) and analysed under the control of Chromeleon™ 7.2 Chromatography Data System. The mobile phase was a mixture of 1.3% NaAc, 0.5% sodium 1-heptanesulfonate, 0.01% EDTA (adjusted to pH 4.0 with 100% acetic acid), 2% methanol (v/v), and 7% Acetonitrile (v/v). All solutions for HPLC analysis were double filtered through 0.2 μm membranes and degassed before use. The flow rate was 1 ml per minute. All chemicals were purchased from Sigma-Aldrich.

### Protein extraction and western blotting

Brains were quickly removed after decapitation. Striatum and midbrain samples were obtained at 0 °C and subsequently homogenized using Disposable Pellet Mixers with Pestle Motor (VWR). Tissue homogenates were prepared in RIPA buffer; pH 7.5, containing 10% Triton 500 μl, 5 μl aprotinin, PMSF 50 μl, Na_3_VO_4_ 100 μl and NaF 20 μl in 4.32 ml PBS. Extracts were clarified by centrifugation at 4 °C (13,200*g* for 20 min). Supernatants were collected and eluted with RIPA buffer, and the proteins were resolved by SDS-PAGE [45]. The primary antibodies used were: β-Tubulin (#05-661, Millipore), β-Actin (Abcam, ab6276, AC15), HA-Tag (C29F4) (#3724, Cell Signalling), Tyrosine Hydroxylase (MAB318, Sigma Aldrich), α-synuclein (610787, BD Transduction Laboratories™), DAT (MAB369, Merckmillipore), LC3 (5F10, Nanotools Antibodies), Tom20 (D8T4N) (#42406, Cell Signalling), Mitofusin-2 (D2D10) (#9482, Cell Signalling), DRP1 (D6C7) (#8570, Cell Signalling), Phospho-Tau Ser202/Thr205 (AT8) (#MN1020, ThermoFisher), and Dopamine D2 Receptor (AB5084P, Merckmillipore). The rabbit anti-mouse (GE healthcare UK, NA934V), or mouse anti-rabbit (GE health UK, NA931V) were used to react with the corresponding primary antibodies. Immunoreactive bands were visualized by enhanced chemiluminescence (GE Healthcare). Densitometry analysis on the bands was calculated using ImageJ software.

### ARG evaluation of [^3^H]FE-PE2I binding

In vitro ARG evaluation was performed on striatal sections. NTg (n = 3) and Tg (n = 3) mice were sacrificed by CO_2_, and mice brains were freshly dissected and frozen in − 80 °C. 10 µm thick brain sections containing the dorsal part of striatum were cut and thaw-mounted on Superfrost Plus glass slides then stored at -80 °C until ARG was performed.

ARG of the dopamine transporter was carried out using [^3^H]FE-PE2I as a radioligand. Sections were pre-incubated for 15 min in 50 mM Tris–HCl buffer, pH 7.4, containing 120 mM NaCl, 5 mM KCl, 2 mM CaCl_2_ and 1 mM MgCl_2_. Incubations were carried out for 1 h in 50 mM Tris–HCl buffer, pH 7.4, containing 120 mM NaCl, 5 mM KCl, 2 mM CaCl_2_, 1 mM MgCl_2_ and 1 nM [^3^H]FE-PE2I (specific radioactivity, 39 Ci/mmol). Non-specific binding was determined in adjacent sections in the presence of 10 μM GBR12909. Sections were washed 3 times (3 min for each wash) in cold (4 ºC) 50 mM Tris–HCl buffer, pH 7.4, followed by a brief dip in distilled water. Radioactivity was detected and quantified using a phosphor imager (scanner: Fuji BAS-5000 image reader; imaging plates: BAS-TR2025, Fujifilm, Tokyo, Japan). The measured photostimulated luminescence (PSL)/mm^2^ values were transformed into radioactivity units and into binding density (pmol/g tissue) based on intensity values obtained using tritium standards (Microscales, American Radiolabeled Chemicals Inc.). Regional specific binding was calculated by subtracting nonspecific binding, as defined in the presence of 10 μM GBR12909, from the total [^3^H]FE-PE2I binding.

### Behavioral analysis

Heterozygous VPS35 D620N mice were crossed with homozygous Nestin cre mice which gives rise to progeny that are 50% Tg and 50% NTg for VPS35 D620N, with both groups of mice carrying the Nestin cre gene. Genotype of the animals was determined by performing PCR on tail samples. Mice were marked randomly at the ears and were tested in all experiments in the same order according to their identification number (number/cage). The experiments and analysis were always performed blind of the genotype. All efforts were made to minimize suffering of the 20-months old Tg and NTg mice. Only one test was performed per day, ranging from 9∶00 AM to 5∶00 PM. On the testing day, animals were put in the test room at least 20 min before testing in order to acclimatize. The entire behavior assay took 2 months. The details of the behavioral tests are described below:

#### Open field

Mice were allowed to explore freely in an arena of 50 × 50 × 50 cm for 15 min. Each mouse was gently placed in the middle of cleaned grey color perspex chamber at the start of the test session, was allowed to explore the arena undisturbed for 15 min, and then removed. The arena was cleaned with 70% ethanol between animals. Video analysis and data acquisition were performed with ANY-maze™ video tracking system (Stoelting, USA) to analyse total distance, total movement duration, moving speed, average speed, and duration at the periphery and center zone.

#### Rotarod

Mice were placed on an accelerating rotarod for a maximum of 5 min (4 to 40 rpm, 5 min ramp, UgoBasile, Italy). The latency to fall from the rotating rod was taken over 3 days, with 2 trials per day and an inter-trial period of 2 h. The last 2 trials of the 3rd day served as a mean value for locomotor abilities. Immediately after each session, the apparatus was cleaned with 70% ethanol.

#### Elevated plus maze

The 53 cm high grey colour high-tech metal alloy maze apparatus, consisted of two open arms (80 × 5 cm) and two closed arms (80 × 5 cm; surrounded by 15-cm-high walls) arranged in a plus shape. The central platform (5 × 5 cm) served as the convergence site of the four arms. A video camera was attached above the set-up to automatically record each trial. Mice were gently placed on the central platform facing a closed arm and were allowed to freely explore the maze for 5 min. The apparatus was cleaned immediately after each session with cotton pads wetted with 70% ethanol. The test was automatically analysed with the ANY-maze™ videotracking system (Stoelting, USA).

#### Tail suspension test

Mice were individually suspended by the tail to a horizontal wooden bar 40 cm above the bench top using an adhesive tape placed approximately 1 cm from the tip of the tail. Typically, mice demonstrate several escape-oriented behaviors interspersed with temporally increasing bouts of immobility. The behaviors were videotaped throughout the 6-min test and the immobility time, defined as lack of all movement except for whisker movement and respiration, was measured with a stopwatch.

### Statistical analysis

The behaviors of the mice were analysed according to the test performed: the activity was automatically recorded in the open field and elevated plus maze by the ANY-Maze video tracking system (Stoelting, USA), or displayed automatically on the rotarod apparatus (UgoBasile, Italy). Data in the tail suspension test and forced swimming test were collected manually with a stopwatch. Statistics were performed using SPSS Software. Two kinds of analysis were usually done: (i) Analysis of variances by two-way ANOVA followed by post-hoc comparisons (Bonferroni), or the unpaired Student’s *t* test for unequal variance. Equality of variances was assessed by Levene’s Test. Significance level was set at *P < 0.05, **p < 0.01, ***p < 0.001.

## Supplementary Information

**Additional file 1: Figure S1.** Body weight analysis of 20-months-old mice. **(A)** Body weight analysis. The body weight of mice was measured and analysed in order to determine if there were variations that could have affected the behavior results. There was no significant difference observed in the body weight (g) (mean ± SEM) among all 4 groups: NTg Male (n = 7), Tg Male (n = 5), NTg Female (n = 7), and Tg Female (n = 7); two-way ANOVA with Bonferroni post hoc test. (NTg Male—Tg Male: P = 0.983, NTg Male—NTg Female: P = 0.999, Tg Male—Tg Female: P = 0.992, NTg Female—Tg Female: P = 0.997).

**Additional file 2: Figure S2.** Assessment of interaction between VPS35 D620N and exogenous oxidative challenge. SH-SY5Y cells were transfected with VPS35 D620N and subjected to 20 µM H_2_O_2_ treatment. **(A)** Trypan Blue Assay. As expected, a significant increase in cell death (normalized against Control 0 h group) was observed in both the Control and VPS35 D620N transfected cells after 24 h of H_2_O_2_ treatment, compared to 0 h of H_2_O_2_ treatment; n = 3, *p < 0.05, **p < 0.01, two-way ANOVA with Bonferroni post hoc test. However, there was no significant difference between Control and VPS35 D620N transfected cells after 24 h of H_2_O_2_ treatment. This suggests that VPS35 D620N does not confer resistance towards exogenous oxidative stress; n = 3, two-way ANOVA with Bonferroni post hoc test. (Control 0 h—D620N 0 h: P = 0.197, Control 0 h – Control 24 h: P = 0.030, D620N 0 h—D620N 24 h: P = 0.002, Control 24 h—D620N 24 h: P = 0.907). **(B)** HPLC analysis of HVA. There was no significant difference observed in HVA level per 10^6^ cells (normalized against Control 0 h group) across all 4 groups: Control 0 h (n = 3), D620N 0 h (n = 3), Control 24 h (n = 3), D620N 24 h (n = 3); two-way ANOVA with Bonferroni post hoc test. (Control 0 h—D620N 0 h: P = 0.934, Control 0 h – Control 24 h: P = 0.999, D620N 0 h—D620N 24 h: P = 0.708, Control 24 h—D620N 24 h: P = 0.666). **(C)** HPLC analysis of DOPAC. There was no significant difference observed in DOPAC level per 10^6^ cells (normalized against Control 0 h group) among all 4 groups: Control 0 h (n = 3), D620N 0 h (n = 3), Control 24 h (n = 3), D620N 24 h (n = 3); two-way ANOVA with Bonferroni post hoc test. (Control 0 h—D620N 0 h: P = 0.839, Control 0 h – Control 24 h: P = 0.999, D620N 0 h—D620N 24 h: P = 0.998, Control 24 h—D620N 24 h: P = 0.741).

## Data Availability

All data generated and analyzed during the current study are included in this published article and its supplementary files.

## Abbreviations

AMPARs: AMPA-type glutamate receptors
ARG: Autoradiography
AT8: Phospho-Tau Ser202/Thr205
DA: Dopamine
DAT: Dopamine transporter
DD2R: Dopamine D2 receptor
DOPAC: 3,4-Dihydroxyphenylacetic acid
DRD1: Dopamine D1 receptor
HVA: Homovanillic acid
LSL: LoxP-flanked stop sequence
MDVs: Mitochondrial-derived vesicles
Mfn2: Mitofusin-2
NTg: Nontransgenic
PD: Parkinson’s disease
SN: Substantia nigra
SNpc: Substantia nigra pars compacta
Tg: Transgenic
TH: Tyrosine hydroxylase
VPS35: Vacuolar protein sorting 35
WT: Wild-type

## References

[1] DeMaagd G, Philip A (2015). Parkinson's disease and its management: part 1: disease entity, risk factors, pathophysiology, clinical presentation, and diagnosis. P T.

[2] Rizzo G, Copetti M, Arcuti S, Martino D, Fontana A, Logroscino G (2016). Accuracy of clinical diagnosis of Parkinson disease: a systematic review and meta-analysis. Neurology.

[3] Reichmann H, Brandt MD, Klingelhoefer L (2016). The nonmotor features of Parkinson's disease: pathophysiology and management advances. Curr Opin Neurol.

[4] Williams ET, Moore DJ (2018). Deciphering the role of VPS35 in Parkinson's disease. J Neurosci Res.

[5] Wang HS, Toh J, Ho P, Tio M, Zhao Y, Tan EK (2014). In vivo evidence of pathogenicity of VPS35 mutations in the Drosophila. Mol Brain.

[6] Ando M, Funayama M, Li Y, Kashihara K, Murakami Y, Ishizu N (2012). VPS35 mutation in Japanese patients with typical Parkinson's disease. Mov Disord.

[7] Kumar KR, Weissbach A, Heldmann M, Kasten M, Tunc S, Sue CM (2012). Frequency of the D620N mutation in VPS35 in Parkinson disease. Arch Neurol.

[8] Deng H, Gao K, Jankovic J (2013). The VPS35 gene and Parkinson's disease. Mov Disord.

[9] Hierro A, Rojas AL, Rojas R, Murthy N, Effantin G, Kajava AV (2007). Functional architecture of the retromer cargo-recognition complex. Nature.

[10] Cullen PJ, Korswagen HC (2011). Sorting nexins provide diversity for retromer-dependent trafficking events. Nat Cell Biol.

[11] Dhungel N, Eleuteri S, Li LB, Kramer NJ, Chartron JW, Spencer B (2015). Parkinson's disease genes VPS35 and EIF4G1 interact genetically and converge on alpha-synuclein. Neuron.

[12] Linhart R, Wong SA, Cao J, Tran M, Huynh A, Ardrey C (2014). Vacuolar protein sorting 35 (Vps35) rescues locomotor deficits and shortened lifespan in Drosophila expressing a Parkinson's disease mutant of Leucine-Rich Repeat Kinase 2 (LRRK2). Mol Neurodegener.

[13] Tsika E, Glauser L, Moser R, Fiser A, Daniel G, Sheerin UM (2014). Parkinson's disease-linked mutations in VPS35 induce dopaminergic neurodegeneration. Hum Mol Genet.

[14] Ishizu N, Yui D, Hebisawa A, Aizawa H, Cui W, Fujita Y (2016). Impaired striatal dopamine release in homozygous Vps35 D620N knock-in mice. Hum Mol Genet.

[15] Simpson EM, Linder CC, Sargent EE, Davisson MT, Mobraaten LE, Sharp JJ (1997). Genetic variation among 129 substrains and its importance for targeted mutagenesis in mice. Nat Genet.

[16] Gelb DJ, Oliver E, Gilman S (1999). Diagnostic criteria for Parkinson disease. Arch Neurol.

[17] Auyeung M, Tsoi TH, Mok V, Cheung CM, Lee CN, Li R (2012). Ten year survival and outcomes in a prospective cohort of new onset Chinese Parkinson's disease patients. J Neurol Neurosurg Psychiatry.

[18] Blonder LX, Slevin JT (2011). Emotional dysfunction in Parkinson's disease. Behav Neurol.

[19] Martinez-Martin P, Damian J (2010). Parkinson disease: depression and anxiety in Parkinson disease. Nat Rev Neurol.

[20] Bichler Z, Lim HC, Zeng L, Tan EK (2013). Non-motor and motor features in LRRK2 transgenic mice. PLoS ONE.

[21] Cataldi S, Follett J, Fox JD, Tatarnikov I, Kadgien C, Gustavsson EK (2018). Altered dopamine release and monoamine transporters in Vps35 p.D620N knock-in mice. NPJ Parkinson's Dis..

[22] Chen X, Kordich JK, Williams ET, Levine N, Cole-Strauss A, Marshall L (2019). Parkinson's disease-linked D620N VPS35 knockin mice manifest tau neuropathology and dopaminergic neurodegeneration. Proc Natl Acad Sci USA.

[23] Zhou ZD, Refai FS, Xie SP, Ng SH, Chan CH, Ho PG (2014). Mutant PINK1 upregulates tyrosine hydroxylase and dopamine levels, leading to vulnerability of dopaminergic neurons. Free Radical Biol Med.

[24] Tang F-L, Erion JR, Tian Y, Liu W, Yin D-M, Ye J (2015). VPS35 in dopamine neurons is required for endosome-to-golgi retrieval of Lamp2a, a receptor of chaperone-mediated autophagy that is critical for α-synuclein degradation and prevention of pathogenesis of Parkinson's disease. J Neurosci.

[25] Olszewska DA, McCarthy A, Lynch T (2016). Commentary: Parkinson's disease genes VPS35 and EIF4G1 interact genetically and converge on alpha-synuclein. Front Neurosci.

[26] Mohan M, Mellick GD (2017). Role of the VPS35 D620N mutation in Parkinson's disease. Parkinsonism Relat Disord.

[27] Munsie LN, Milnerwood AJ, Seibler P, Beccano-Kelly DA, Tatarnikov I, Khinda J (2015). Retromer-dependent neurotransmitter receptor trafficking to synapses is altered by the Parkinson's disease VPS35 mutation p.D620N. Hum Mol Genet..

[28] Wang C, Niu M, Zhou Z, Zheng X, Zhang L, Tian Y (2016). VPS35 regulates cell surface recycling and signaling of dopamine receptor D1. Neurobiol Aging.

[29] Wu S, Fagan RR, Uttamapinant C, Lifshitz LM, Fogarty KE, Ting AY (2017). The dopamine transporter recycles via a retromer-dependent postendocytic mechanism: tracking studies using a novel fluorophore-coupling approach. J Neurosci.

[30] Williams ET, Chen X, Moore DJ (2017). VPS35, the retromer complex and Parkinson's disease. J Parkinson's Dis.

[31] Braschi E, Goyon V, Zunino R, Mohanty A, Xu L, McBride HM (2010). Vps35 mediates vesicle transport between the mitochondria and peroxisomes. Curr Biol.

[32] Sugiura A, McLelland GL, Fon EA, McBride HM (2014). A new pathway for mitochondrial quality control: mitochondrial-derived vesicles. EMBO J.

[33] Yamamoto H, Itoh N, Kawano S, Yatsukawa Y, Momose T, Makio T (2011). Dual role of the receptor Tom20 in specificity and efficiency of protein import into mitochondria. Proc Natl Acad Sci USA.

[34] Filadi R, Pendin D, Pizzo P (2018). Mitofusin 2: from functions to disease. Cell Death Dis.

[35] Frank S, Gaume B, Bergmann-Leitner ES, Leitner WW, Robert EG, Catez F (2001). The role of dynamin-related protein 1, a mediator of mitochondrial fission, in apoptosis. Dev Cell.

[36] Tanida I, Ueno T, Kominami E (2008). LC3 and autophagy. Methods Mol Biol (Clifton, NJ).

[37] Hall H, Halldin C, Guilloteau D, Chalon S, Emond P, Besnard J (1999). Visualization of the dopamine transporter in the human brain postmortem with the new selective ligand [125I]PE2I. NeuroImage.

[38] Jiang M, Vanan S, Tu HT, Zhang W, Zhang ZW, Chia SY (2020). Amyloid precursor protein intracellular domain-dependent regulation of FOXO3a inhibits adult hippocampal neurogenesis. Neurobiol Aging.

[39] Miura E, Hasegawa T, Konno M, Suzuki M, Sugeno N, Fujikake N (2014). VPS35 dysfunction impairs lysosomal degradation of alpha-synuclein and exacerbates neurotoxicity in a Drosophila model of Parkinson's disease. Neurobiol Dis.

[40] Follett J, Bugarcic A, Yang Z, Ariotti N, Norwood SJ, Collins BM (2016). Parkinson disease-linked Vps35 R524W mutation impairs the endosomal association of retromer and induces alpha-synuclein aggregation. J Biol Chem.

[41] Kasahara Y, Arime Y, Kubo Y, Fukui A, Sora I (2011). Neuronal development of the hyperdopaminergic animal model. Jpn J Psychopharmacol..

[42] Hall FS, Itokawa K, Schmitt A, Moessner R, Sora I, Lesch KP (2014). Decreased vesicular monoamine transporter 2 (VMAT2) and dopamine transporter (DAT) function in knockout mice affects aging of dopaminergic systems. Neuropharmacology.

[43] Chen Z-C, Zhang W, Chua L-L, Chai C, Li R, Lin L (2017). Phosphorylation of amyloid precursor protein by mutant LRRK2 promotes AICD activity and neurotoxicity in Parkinson’s disease. Sci Signal..

[44] Oh SW, Harris JA, Ng L, Winslow B, Cain N, Mihalas S (2014). A mesoscale connectome of the mouse brain. Nature.

[45] Chen Z, Cao Z, Zhang W, Gu M, Zhou ZD, Li B (2017). LRRK2 interacts with ATM and regulates Mdm2-p53 cell proliferation axis in response to genotoxic stress. Hum Mol Genet.

