# Supplementary figures and images for: Altered striatal dopamine levels in Parkinson’s disease VPS35 D620N mutant transgenic aged mice
Source: Mol Brain. 2020 Dec 1;13:164. doi: 10.1186/s13041-020-00704-3 (PMC7706192; doi:10.1186/s13041-020-00704-3)

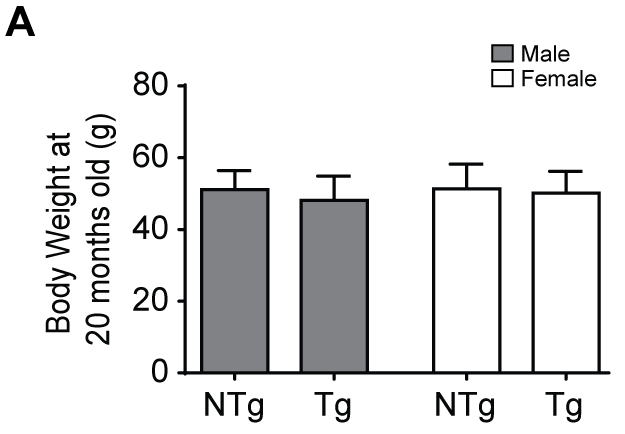

Supplement: Supplementary file 1 — Additional file 1: Figure S1. Body weight analysis of 20-months-old mice. (A) Body weight analysis. The body weight of mice was measured and analysed in order to determine if there were variations that could have affected the behavior results. There was no significant difference observed in the body weight (g) (mean ± SEM) among all 4 groups: NTg Male (n = 7), Tg Male (n = 5), NTg Female (n = 7), and Tg Female (n = 7); two-way ANOVA with Bonferroni post hoc test. (NTg Male—Tg Male: P = 0.983, NTg Male—NTg Female: P = 0.999, Tg Male—Tg Female: P = 0.992, NTg Female—Tg Female: P = 0.997). [file 13041_2020_704_MOESM1_ESM.tif]

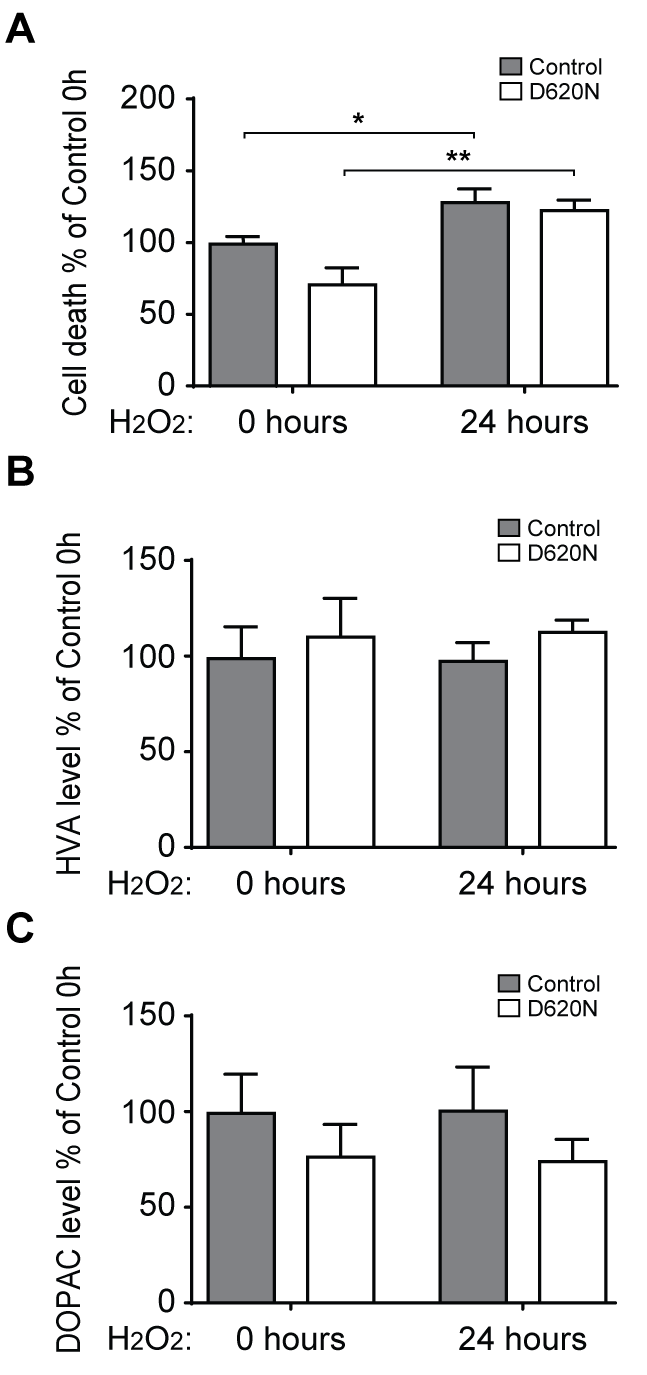

Supplement: Supplementary file 2 — Additional file 2: Figure S2. Assessment of interaction between VPS35 D620N and exogenous oxidative challenge. SH-SY5Y cells were transfected with VPS35 D620N and subjected to 20 µM H2O2 treatment. (A) Trypan Blue Assay. As expected, a significant increase in cell death (normalized against Control 0 h group) was observed in both the Control and VPS35 D620N transfected cells after 24 h of H2O2 treatment, compared to 0 h of H2O2 treatment; n = 3, *p < 0.05, **p < 0.01, two-way ANOVA with Bonferroni post hoc test. However, there was no significant difference between Control and VPS35 D620N transfected cells after 24 h of H2O2 treatment. This suggests that VPS35 D620N does not confer resistance towards exogenous oxidative stress; n = 3, two-way ANOVA with Bonferroni post hoc test. (Control 0 h—D620N 0 h: P = 0.197, Control 0 h – Control 24 h: P = 0.030, D620N 0 h—D620N 24 h: P = 0.002, Control 24 h—D620N 24 h: P = 0.907). (B) HPLC analysis of HVA. There was no significant difference observed in HVA level per 106 cells (normalized against Control 0 h group) across all 4 groups: Control 0 h (n = 3), D620N 0 h (n = 3), Control 24 h (n = 3), D620N 24 h (n = 3); two-way ANOVA with Bonferroni post hoc test. (Control 0 h—D620N 0 h: P = 0.934, Control 0 h – Control 24 h: P = 0.999, D620N 0 h—D620N 24 h: P = 0.708, Control 24 h—D620N 24 h: P = 0.666). (C) HPLC analysis of DOPAC. There was no significant difference observed in DOPAC level per 106 cells (normalized against Control 0 h group) among all 4 groups: Control 0 h (n = 3), D620N 0 h (n = 3), Control 24 h (n = 3), D620N 24 h (n = 3); two-way ANOVA with Bonferroni post hoc test. (Control 0 h—D620N 0 h: P = 0.839, Control 0 h – Control 24 h: P = 0.999, D620N 0 h—D620N 24 h: P = 0.998, Control 24 h—D620N 24 h: P = 0.741). [file 13041_2020_704_MOESM2_ESM.tif]
